# Predictors of COVID-19 Vaccine Intention among the Saudi Arabian Population: A Cross-Sectional Survey

**DOI:** 10.3390/vaccines9080892

**Published:** 2021-08-12

**Authors:** Mohammed Noushad, Mohammad Zakaria Nassani, Pradeep Koppolu, Anas B. Alsalhani, Abdulaziz Samran, Ali Alqerban, Ghadah Salim Abusalim, Ali Barakat, Mashari Bandar Alshalhoub, Samer Rastam

**Affiliations:** 1 Department of Restorative and Prosthetic Dental Sciences, College of Dentistry, Dar Al Uloom University, Riyadh 13313, Saudi Arabia; mznassani@dau.edu.sa (M.Z.N.); asamran@dau.edu.sa (A.S.); ali.ab@dau.edu.sa (A.B.); 1141460@du.edu.sa (M.B.A.); 2 Biomaterials Unit, Health Campus, School of Dental Sciences, Universiti Sains Malaysia, Kubang Kerian 16150, Malaysia; 3 Department of Preventive Dental Sciences, College of Dentistry, Dar Al Uloom University, Riyadh 13313, Saudi Arabia; pradeep@dau.edu.sa (P.K.); a.alqerban@psau.edu.sa (A.A.); 4 Department of Oral Medicine and Diagnostic Sciences, Vision College of Dentistry and Nursing, Vision Colleges, Riyadh 11691, Saudi Arabia; aalsalhani@vision.edu.sa; 5 Department of Prosthodontics, College of Dentistry, Ibb University, Ibb 70270, Yemen; 6 Department of Preventive Dental Sciences, College of Dentistry, Prince Sattam Bin Abdulaziz University, Al-Kharj 11942, Saudi Arabia; 7 Department of Medical Laboratory Science, College of Applied Medical Sciences, Prince Sattam Bin Abdulaziz University, Al-Kharj 11942, Saudi Arabia; g.abusalim@psau.edu.sa; 8 Department of Clinical Sciences, Vision College of Medicine, Vision Colleges, Riyadh 11691, Saudi Arabia; mroustom@vision.edu.sa

**Keywords:** COVID-19, vaccine intention, Saudi Arabia, population immunity

## Abstract

The long-term solution to managing the current COVID-19 pandemic is through mass immunization of the population. However, uncertainty or unwillingness to receive the vaccine could be a barrier in attaining sufficient vaccine coverage. Therefore, understanding the psychology of the population towards the vaccines against COVID-19 is of paramount importance. Our study was aimed at determining the predictors of COVID-19 vaccine intention in the Saudi Arabian population. A structured questionnaire guided by the ‘Report of the SAGE working group on vaccine hesitancy’ was administered during a span of two months among the general population from all administrative regions of Saudi Arabia, proceeding the launch of the vaccination campaign. In total, 879 out of 1600 subjects responded and completed the survey (response rate 54.9%). About 56 percent of the participants intended to be vaccinated. The predictors of a higher intention to vaccinate included those 50 years of age or older, male subjects, people suffering from systemic disease/s, subjects who were not previously infected with COVID-19, those who follow the updates about COVID-19 vaccines, and adults with a higher level of anxiety about contracting coronavirus (*p* < 0.05). Results from our study and other similar studies can aid policy makers and stakeholders in planning effective strategies based on the changing behavior of the population.

## 1. Introduction

The global outbreak of Coronavirus disease 2019, popularly called COVID-19, has resulted in a tremendous loss of lives and widespread socioeconomic disruption throughout the world. As of 22 June 2021, up to 180 million have been infected with COVID-19 and almost 3.9 million have died due to the disease [1]. The pandemic has created a crucial threat to the health of the public along with catastrophic financial consequences. In addition to protective measures, such as social distancing, wearing face masks, the quarantine of infected individuals, etc., an effective vaccine will remain the ideal strategy in not only minimizing the spread of the disease, but also promoting positive socioeconomic and clinical outcomes.

Vaccines are among one of the leading public health accomplishments of the 20th century and the implementation of immunization has contributed greatly against preventable diseases. However, vaccine hesitancy is one emerging challenge that has been classified by the World Health Organization (WHO) as one of the ten main threats to global health [2]. Vaccine hesitancy is a complex problem and the responsible factors influencing this phenomenon are greatly changing across populations. Researchers have associated vaccine hesitancy with several factors, including misconceptions about the necessity for vaccination, the vaccine’s side effects, lack of confidence in the health system, vaccination history, lack of knowledge about the disease and the vaccine, the efficacy and safety of the vaccine, disease severity, and the cost of being vaccinated, if any [3,4,5].

Researchers all over the world have been working tirelessly to develop effective vaccines against COVID-19 [6,7]. As a result, within a year, several vaccines have been granted approval for emergency use. However, the victory of immunization against COVID-19 will depend on sufficient vaccine coverage and uptake rates among the population. Recent research has suggested skepticism and uncertainties about the COVID-19 vaccines as a result of the public’s suspicion of the government, and the spread of misinformation through social media [8,9,10]. In addition to this, similar kinds of fluctuations in intentions of being vaccinated against COVID-19 in France and Australia have been documented [9,11].

COVID-19 is the second widespread disease outbreak to have affected Saudi Arabia within a few years, the first being the Middle East Respiratory Syndrome Coronavirus (MERS-CoV) [12]. Even before the confirmation of the first case of COVID-19 within its borders, Saudi Arabia had taken proactive steps in minimizing the spread of the disease. As of 24 June 2021, Saudi Arabia has about 476,000 confirmed COVID-19 cases and about 7700 related fatalities (WHO Dashboard) [13]. A rapid rollout of the available vaccines through effective vaccination campaigns in order to immunize as many people in a short time remains the top priority. However, misinformation and rumors about vaccines through social media could affect vaccine intention, possibly threatening the response to the present crisis. The outcomes of studies suggest that unwillingness and uncertainty regarding vaccination pose challenges in attaining vaccination coverage to reach herd immunity [14]. Previous research that analyzes the acceptance of a hypothetical vaccine against COVID-19 was carried out in Saudi Arabia before the approval of the vaccines [15]. However, following the approval of the Pfizer-BioNTech and Oxford-AstraZeneca COVID-19 vaccines in Saudi Arabia, and the rapid spread of false information, it is very much important to determine the predictors of vaccine intention amongst the public, as decisions about vaccination are multifactorial and may change over time. Thus, our study focused on determining the predictors of COVID-19 vaccine intention among the Saudi population after the vaccination campaigns in Saudi Arabia gathered sufficient pace. Results from our study can aid policy makers and health officials to make evidence-based communication plans to enhance uptake among the general public.

## 2. Methods

### 2.1. Study Design

A cross-sectional self-administered survey was conducted among the adult general population in Saudi Arabia in the months of February and March 2021. The ‘Report of the SAGE working group on vaccine hesitancy’ was used as a guide in preparing the questionnaire [16]. As part of the validation of the study, a pilot study was initially carried out on 10 participants, after which expert opinions were taken from four specialists in the field. Forms completed as part of the pilot study were not included in the actual study. The survey questionnaire, developed on Google Forms, was distributed using convenience sampling through WhatsApp and Snapchat applications to 1600 prospective participants. The questionnaire required less than 5 min to complete. Participation was voluntary and the participants provided informed consent on the survey platform before proceeding to the survey items. The participants’ anonymity was guaranteed during the data collection process. All targeted participants were reminded once, during the survey period.

This study was approved by the Research Committee of College of Dentistry, Dar Al Uloom University, Riyadh, Saudi Arabia (COD/IRB/2020/2).

### 2.2. Sample

The power of the sample size was calculated using Open Source Epidemiologic Statistics for Public Health—OpenEpi (http://www.openepi.com/Menu/OE_Menu.htm, accessed on 15 May 2021). The current study used the “Sample Size for % Frequency in a Population” module, with 50% as anticipated frequency that is recommended for unknown frequency, and 5% absolute precision. The result was a sample size of 664 to obtain a 99% confidence interval, which was our target in the current study.

Participants included the general population from all the administrative regions of Saudi Arabia, representative of the population under study. Healthcare workers were excluded from the study.

Participants below the age of 18 years were not included in the study. Participants were not paid compensation for participation in the study. The survey form was designed in such a way that only complete forms would qualify for submission.

### 2.3. Measures

#### 2.3.1. Trust in Vaccines, Vaccine Manufacturers, and Health Authorities

General attitudes towards vaccines were measured using a 6-item scale. First, participants were asked if vaccines were really necessary to overcome the pandemic. Three assessment questions on perceived trust in vaccine manufacturers included whether participants trusted vaccines of only specific companies, and whether vaccine manufacturers followed recommended development and production guidelines. Responses were rated on a five-point Likert scale from 1 “strongly agree” to 5 “strongly disagree”. Participants’ attitudes towards the health care system were measured using a 2-item scale. Participants were asked if they were happy with the health authorities’ handling of the pandemic, and their management of vaccination campaigns. Responses were rated on a five-point Likert scale from 1 “strongly agree” to 5 “strongly disagree”.

#### 2.3.2. Intention to Vaccinate

This was measured using a 7-item scale. First, participants were asked if vaccines should be made mandatory and if the participant intended to be vaccinated. Further questions included fear of the vaccine, care for others who would be in greater need for the vaccine, and intention to protect others with weaker immunity. Responses were rated on a five-point Likert scale from 1 “strongly agree” to 5 “strongly disagree”.

### 2.4. Predictor Variables

Socio-demographic factors included age group, sex, nationality, region, and employment. Participants’ reports on chronic medical conditions (e.g., asthma, diabetes, hypertension, heart disease, and/or cancer) were used to indicate the presence or absence of pre-existing co-morbidity. Other variables included participants’ self-updating on COVID-19 vaccine development, prior infection with COVID-19, perception of COVID-19 severity, compliance with government COVID-19 guidelines, and anxiety towards contracting COVID-19.

### 2.5. Statistical Analysis

Descriptive statistics were expressed as percentages and numbers for each item/survey question. The main outcome of this study was the intention to vaccinate. The current study considered any participant to have an intention to vaccinate if he/she agreed or strongly agreed on the item “I will get vaccinated with the Covid-19 vaccine”, or if they had already taken the vaccine. Bivariate statistical analysis of the relationship between the main outcome “intention to vaccinate” and predictors was performed using the Chi-squared test for trend for ordinal factors, and the Chi-squared test for categorical variables. A multivariate binary logistic regression model was used to determine the predictors for intention to vaccinate. The following factors were examined as potential predictors for intention to vaccinate: age group, sex, nationality, presence of any medical condition, previous infection with COVID-19, following updates on the development of vaccines against COVID-19, opinion about the severity of COVID-19, compliance with COVID-19 preventive guidelines, and anxiety about contracting COVID-19. We used the Bonferroni correction to overcome the possibilities of false positive results as a result of multiple comparisons. Based on this adjustment, the significance level was set at *p* < 0.0056. All statistical analyses were performed using IBM SPSS Statistics version 25.0 (IBM Corp. Released 2017. IBM SPSS Statistics for Windows, Version 25.0. Armonk, NY, USA: IBM Corp). 

## 3. Results

Demographics and characteristics of the study population are presented in Table 1. In total, 879 out of 1600 subjects responded and completed the survey (response rate 54.9%). Table 2 shows that almost 16% of the respondents were previously infected with COVID-19. Of the participants, only 11.4% were vaccinated against COVID-19 at the time of the survey. A high proportion (79.6%) of the participants have been updating themselves about the development of COVID-19 vaccines. In terms of the severity of COVID-19, almost half of the sample rated it as a severe disease (50.4%) and 68% expressed their compliance with COVID-19 preventive guidelines. Approximately a fifth (19.3%) of the participants indicated high anxiety levels about contracting COVID-19 (Table 2).

While a clear majority (66.9%) of the study population agreed that vaccines against COVID-19 are important to overcome the pandemic and bring life back to normal, trust in the available vaccines was less than optimal, with almost 36% of the participants indicating trust in vaccines of only certain companies and 51% being not sure. Some doubts about the vaccines were also expressed by a considerable proportion of the respondents as 32% thought that the vaccines had been produced in a hurry without following recommended guidelines. On the level of trust in the health authorities’ overall management of the COVID-19 pandemic, a positive attitude was predominant among the study group. The above results are summarized in Table 3.

Support for a mandatory vaccination program against COVID-19 was shown by around 55% of the participants. Additionally, 56% indicated their intention to be vaccinated/have been vaccinated. On the contrary, 32% of the study sample indicated their fear of taking the vaccine. Awareness regarding the importance of taking the vaccine as a means of protecting vulnerable people was high (70%). In addition, more than half of the participants (55.1%) expressed their readiness to delay taking the vaccine in favor of people who are in greater need of vaccination. Table 4 illustrates the abovementioned findings.

The bivariate statistical analysis indicated an association between participants’ intention to vaccinate and eight factors (*p* < 0.0056) (Table 5). It can be noted that the intention to vaccinate increased significantly with older age and in participants with systemic disease/s. Moreover, male subjects showed a significantly higher intention to vaccinate than females. For those who were previously infected with COVID-19, the intention to take the vaccine was significantly lower. As well, there has been an association between the intention to vaccinate and updating oneself on the development of COVID-19 vaccines/perception about the severity of COVID-19. Greater compliance with COVID-19 preventive guidelines and a higher level of anxiety about contracting COVID-19 were associated with a greater intention to vaccinate.

The logistic regression analysis indicated six predictors for the intention to vaccinate among the adult population in Saudi Arabia. Higher intention to vaccinate can be predicted among participants 50 years of age or older, male subjects, people suffering from systemic disease/s, subjects who were not previously infected with COVID-19, those who follow the updates about COVID-19 vaccines and adults with higher levels of anxiety about contracting coronavirus (*p* < 0.05) (Table 6).

## 4. Discussion

Following the approval of COVID-19 vaccines for emergency use in December 2020, Saudi Arabia was one of the first countries to launch its vaccination campaign against COVID-19. The Minister of Health took the first jab, reassuring the public on the safety of the vaccine and urging them to register for it free of cost [17]. Since Saudi Arabia receives millions of pilgrims year-round, a robust vaccination campaign becomes all the more important. As a guide to achieving maximum vaccine coverage, our study aimed at understanding the predictors of COVID-19 vaccine intention among the general population in Saudi Arabia. 

In our study, 56.2 percent of the participants had either taken the vaccine or agreed to do so. On the positive side, only 12 percent refused to take the vaccine, the remaining 31 percent being undecided. The percentage of those willing to be vaccinated in our study is less than that of a previous study, in which 71.9 percent of the respondents agreed to be vaccinated against COVID-19 [18]. The reasons could be the difference in the study population, geographical distribution of participants, and the study period. The current study was conducted at a time when the controversy of blood clots associated with the Oxford-AstraZeneca vaccine was gathering pace [19]. Moreover, the previous study also included healthcare workers (27.5 percent), and the percentage of males and Saudis was higher, all indicators of higher intention to be vaccinated. However, compared to another similar study, in which only about 48 percent of the participants intended to be vaccinated, the acceptance rate in our study is higher [20]. 

Similar to our study, several studies in Saudi Arabia and other countries have suggested being male as a predictor for greater intention of being vaccinated for COVID-19 [18,21]. A study conducted in Saudi Arabia just before the launch of the vaccination campaign indicated that females were less likely to accept the vaccine as compared to males [20]. Similar results were reported in other studies in Saudi Arabia that were conducted after the launch of the vaccination campaign [22,23]. From a global perspective, a systemic review of COVID-19 vaccine intention in 33 countries indicated that males were more likely to accept the vaccine [24]. A report in the local newspaper indicating a lower-than-expected turnout for vaccination among females in spite of infections being higher among them confirms our results [25]. The higher mortality rate among males due to COVID-19 could be a reason for this variation.

Studies have shown that the intention to vaccinate against COVID-19 is directly proportional to its severity perception [26,27]. Participants in our study who perceived COVID-19 to be severe were most likely to be vaccinated, with those who perceived it to be a mild disease being least likely. As reported earlier, our study also suggested that a positive outlook towards complying with government-implemented preventive guidelines was also apparent in their greater intention to be vaccinated [14]. A study in neighboring Kuwait indicated similar results [28].

We performed multivariate logistic regression analysis to pinpoint possible predictors of vaccine intention in the general population in Saudi Arabia. Advancing age, being male, presence of comorbidities, previous infection with COVID-19, updating oneself on the development of COVID-19 vaccines, and anxiety about contracting COVID-19 were significantly associated with the willingness to be vaccinated against COVID-19. In agreement with other studies, the intention to be vaccinated was greatest in the oldest age group in our study as well, with the intention decreasing with decreasing age [15]. Participants in our study with one or more comorbidities were more likely to be vaccinated than those without. This is a logical finding, as individuals with comorbidities are more likely to require critical care or die from COVID-19 than those without. This has been reported in other studies as well [18,29].

The intention to be vaccinated was lower among those who had contracted COVID-19, which could be a result of their safety perceptions due to the natural immunity acquired after contracting the disease. Similar to our study, an exploratory descriptive study in India concluded that doctors who updated their knowledge on COVID-19 vaccines based on current research and government policies had a positive outlook towards vaccines. The same was reported to be true for other hospital staff who informed themselves based on information from doctors and paramedics [30]. We also found a correlation between the anxiety about contracting COVID-19 and the intention to be vaccinated. A previous study before the start of the vaccination campaign in Saudi Arabia and other studies have also reported that participants who were concerned about becoming infected were 2.13 times more likely to take the vaccine [15,31].

An interesting finding in our study is that about 35 percent of the participants agreed that they trust vaccines of only certain companies, which could contribute to their intention to take the vaccine. The reason for this could be the negative social media coverage on the rare blood clots associated with patients receiving the Oxford-AstraZeneca vaccine. Since the survey period, public health education through scientific and newspaper publications, social media, etc., on the rarity of the blood clots and the importance of vaccination to protect oneself and the community could have positively changed the public perception towards the concerned vaccines. This is evident from the fact that, as of 24 June 2021, more than 16.8 million doses have been administered through 587 vaccination centers covering 70 percent of the total adult population in Saudi Arabia [32]. This could also indicate a change in public perception towards the COVID-19 vaccines, which is expected to change over time. Moreover, it highlights the importance of studies on vaccine intention in identifying vulnerable communities and individuals, and the factors contributing to their vulnerability.

Even though only 56 percent of the participants in our study agreed to be vaccinated against COVID-19, some findings suggest a positive future outlook. For example, 55 percent of the participants agreed to delaying taking the vaccine in favor of people who are in greater need, and 70 percent agreed that being vaccinated was important for protecting people with a weaker immune system. These findings suggest that the rate of vaccine intention may increase over time as more vaccines are procured, and accessibility increases.

The high percentage of participants in our study who are undecided about being vaccinated compared to those who disagree is a positive indication as it would be easier to convince them to take the vaccine than those who disagree. Results from our study and other similar studies coupled with the great amount of trust in authorities (about 90 percent) among the participants will aid policymakers in targeting vulnerable categories and individuals to attain immunization sufficient to attain population immunity.

Our study has a few strengths. The study included participants from all major regions of Saudi Arabia representing different demographic characteristics. Although our results are not representative of the population at large, they do serve as a guide to policymakers and stakeholders. The major limitation of our study is the mode of the study. Since we used a web-based self-administration mode of survey, there could be potential bias among the participants in responding to the survey questions. However, due to the restrictions related to the pandemic, this was the best mode currently available. Moreover, there is a possibility that individuals who disagreed to participate are hesitant to take the vaccine. Since the rate of intention of the population could change over time, further studies will provide added value on the evolving vaccine intention trend among the public in Saudi Arabia.

## 5. Conclusions

While about 56 percent of the participants in our study intended to be vaccinated, only 12 percent refused to do so, with the rest being undecided. The significant predictors of intention to vaccinate included advancing age, being male, presence of comorbidities, previous infection with COVID-19, updating oneself on the development of COVID-19 vaccines and anxiety about contracting COVID-19. Our study was carried out at a time when there was negative coverage of the AstraZeneca vaccine, based on reports of rare blood clots associated with the vaccine. Since then, massive global information campaigns have been able to educate the population on the importance of being vaccinated. As the vaccination campaign gathers pace, the behavior of the public is expected to change. Therefore, results from this study and other similar studies will aid policymakers in tailoring vaccination campaigns based on the behavior of the public. The experience Saudi Arabia gained in managing the MERS-CoV epidemic could also be an added advantage to plan effective public health strategies to promote sufficient vaccine uptake to achieve population immunity.

## Figures and Tables

**Table 1:** Characteristics of participants (No = 879).

**Age (Years)**		**Sex**		**Nationality**	
18–29 y	461(52.4%)	Male	271(30.8%)	Saudi	740 (84.2%)
30–49 y	351(39.9%)				
50–64 y	61(6.9%)	Female	608(69.2%)	Non-Saudi	139 (15.8%)
Above 64	6(0.7%)				
**Location in Saudi Arabia**		**Employment**		**Medical Condition**	
Northern Region	31(3.5%)	Employed	430(48.9%)	Healthy	754(85.8%)
Southern Region	136(15.5%)				
Central Region	467(53.1%)	Unemployed	449(51.1%)	Has systemic disease/s	125(14.2%)
Eastern Region	150(17.1%)				
Western Region	95(10.8%)				

**Table 2:** General questions regarding COVID-19.

No	Question	Participants’ Response *n (%)*
1	Have you had COVID-19?	Yes 139 (15.8) No 740 (84.2)
2	Have you taken the COVID-19 vaccine?	Yes 100 (11.4) No 779 (88.6)
3	Have you been updating yourself on the development of vaccines against COVID-19?	Yes 700 (79.6) No 179 (20.4)
4	In your opinion, how would you rate the severity of COVID-19 disease?	Mild 38 (4.3) Moderate 398 (45.3) Severe 443 (50.4)
5	How would you rate your compliance with COVID-19 preventive guidelines?	Good 600 (68.3) Moderate 261 (29.7) Poor 18 (2)
6	To what extent are you anxious about contracting (getting infected with) COVID-19?	High 170 (19.3) Moderate 464 (52.8) Low 245 (27.9)

**Table 3:** Trust in COVID-19 vaccines and health authorities.

No	Statement	Participants’ Response *n (%)*				
		Strongly Agree	Agree	Not Sure	Disagree	Strongly Disagree
1	Vaccines are necessary to overcome the COVID-19 pandemic and get back to normal life.	324(36.9)	264(30)	252(28.7)	17(1.9)	22(2.5)
2	I trust COVID-19 vaccines of ONLY certain companies.	117(13.3)	197(22.4)	444(50.5)	65(7.4)	56(6.4)
3	I think that vaccines against COVID-19 have been produced in a hurry without following recommended clinical trials and approval guidelines.	96(10.9)	184(20.9)	358(40.7)	152(17.3)	89(10.1)
5	I am happy with the way the health authorities have been managing the COVID-19 pandemic so far.	483(54.9)	305(34.7)	60(6.8)	20(2.3)	11(1.3)
6	I am happy with the health authorities’ efficient organization of the COVID-19 vaccination campaigns through the digital applications and other methods.	475(54)	294(33.4)	82(9.3)	15(1.7)	13(1.5)

**Table 4:** Intention to vaccinate.

No	Statement	Participants’ Response *n (%)*				
		Strongly Agree	Agree	Not Sure	Disagree	Strongly Disagree
1	I support a mandatory vaccination program for COVID-19.	297(33.8)	185(21)	201(22.9)	116(13.2)	80(9.1)
2	I will get vaccinated with the COVID-19 vaccine.	308(35)	186(21.2)	278(31.6)	52(5.9)	55(6.3)
3	I will wait for other people to take the COVID-19 vaccine, as I am afraid to take it myself.	88(10)	197(22.4)	206(23.4)	181(20.6)	117(13.3)
4	I will delay taking the COVID-19 vaccine, as I feel there are others who deserve it more than me.	165(18.8)	319(36.3)	191(21.7)	83(9.4)	38(4.3)
5	Getting myself vaccinated for COVID-19 is important because I can also protect people with a weaker immune system.	321(36.5)	294(33.4)	207(23.5)	32(3.6)	25(2.8)

**Table 5:** Bivariate statistical analysis of relationship between the main outcome “intention to vaccinate” and potential predictors.

All Participants	*% (n)*	*p*
	56.2% (494/879)	
**Age**		0.001 *
18–29 years	52.3% (241/461)	
30–49 years	57.5% (202/351)	
50 years or above	76.1% (51/67)	
**Sex**		<0.001 **
Male	65.3% (177/271)	
Female	52.1% (317/608)	
**Nationality**		0.02
Saudi	57.8% (428/740)	
Non-Saudi	47.5% (66/139)	
**Medical condition**		<0.001 **
Healthy	53.6% (404/754)	
Has systemic disease/s	72.0% (90/125)	
**Previously infected with COVID-19**		0.001 **
No	58.6% (434/740)	
Yes	43.2% (60/139)	
**Updating self on the development of vaccines against COVID-19**		0.001 **
No	41.9% (75/179)	
Yes	59.9% (419/700)	
**Opinion about the severity of COVID-19**		<0.001 *
Mild	44.7% (17/38)	
Moderate	49.2% (196/398)	
Severe	63.4% (281/443)	
**Compliance with COVID-19 preventive guidelines**		0.004 *
Poor	38.9% (7/18)	
Moderate	50.2% (131/261)	
Good	59.3% (356/600)	
**Anxiety about contracting COVID-19**		<0.001 *
Low	46.5% (114/245)	
Moderate	57.5% (267/464)	
High	66.5% (113/170)	

*: *p* was calculated using Chi-square test for trend. **: *p* was calculated using Chi-square test; significant difference was set at *p* < 0.0056 using the Bonferroni correction for multiple comparisons.

**Table 6:** Predictors of intention to vaccinate.

All Participants	Odds Ratio (95% Confidence Interval)	*p*
**Age**		
18–29 years	Ref	
30–49 years	1.05 (0.78–1.42)	0.73
50 years or above	2.5 (1.32–4.74)	0.005 *
**Sex**		
Male	Ref	
Female	0.52 (0.38–0.72)	<0.001 *
**Nationality**		
Saudi	Ref	
Non-Saudi	0.71 (0.48–1.05)	0.09
**Medical condition**		
Healthy	Ref	
Has systemic disease/s	1.66 (1.06–2.6)	0.03 *
**Previously infected with COVID-19**		
No	Ref	
Yes	0.61 (0.41–0.9)	0.012 *
**Updating self on the development of vaccines against COVID-19**		
No	Ref	
Yes	1.81 (1.27–2.59)	0.001 *
**Opinion about the severity of COVID-19**		
Mild	Ref	
Moderate	0.87 (0.42–1.81)	0.71
Severe	1.25 (0.6–2.62)	0.55
**Compliance with COVID-19 preventive guidelines**		
Poor	Ref	
Moderate	1.17 (0.38–3.55)	0.79
Good	1.5 (0.49–4.56)	0.48
**Anxiety about contracting COVID-19**		
Low	Ref	
Moderate	1.45 (1.03–2.04)	0.034 *
High	1.84 (1.18–2.88)	0.008 *

Odds ratio and 95% confidence interval was calculated by a multivariate binary logistic model; * Significant difference at *p* < 0.05.

## Data Availability

The questionnaire used in the current study is not publicly available due to certain restrictions. However, it is available from the corresponding author (Mohammed Noushad) on reasonable request.
